# Draft Genome Sequences of *Ralstonia solanacearum* Race 3 Biovar 2 Strains with Different Temperature Adaptations

**DOI:** 10.1128/genomeA.00815-15

**Published:** 2015-08-13

**Authors:** Kat (Xiaoli) Yuan, Jeff Cullis, C. André Lévesque, James Tambong, Wen Chen, Christopher T. Lewis, Solke H. De Boer, Xiang (Sean) Li

**Affiliations:** a Canadian Food Inspection Agency, Charlottetown Laboratory (CFIA-CL), Charlottetown, Canada; b Agriculture and Agri-Food Canada (AAFC), Ottawa, Canada

## Abstract

*Ralstonia solanacearum* race 3 biovar 2 (R3bv2) causes brown rot of potato in countries with temperate climates. Here, we report two draft genome sequences of *R. solanacearum* R3bv2 NCPPB909 and CFIA906 with different temperature adaptations. Analysis of these genome sequences will provide detailed insight on virulence, functionality, and plant/pest interactions of this widely distributed and regulated pathogen.

## GENOME ANNOUNCEMENT

For more than a century, *Ralstonia solanacearum* (Smith, 1896) Yabuuchi et al. (1) species complex has been one of the most economically important phytopathogenic bacteria because of its lethality, complex host profile, and worldwide distribution. This bacterium causes vascular wilt in more than 200 plant species belonging to over 54 families in tropical and subtropical regions (2, 3). *R. solanacearum* race 3 biovar 2 (R3bv2) strains, which cause brown rot and bacterial wilt of potato, southern wilt of geranium, and bacterial wilt of tomato and other solanaceous crops, were classified as phylotype II sequevars 1 and 2. Different from other strains of the *R. solanacearum* species complex, R3bv2 strains are adapted to a temperate climate and have caused significant losses to the potato industry throughout Europe during the last decade. Latently infected geranium cuttings from Kenya and Central America were believed to be the cause of substantial damage in greenhouse-grown crops in Belgium, Germany, the Netherlands, and the United States (4, 5). So far, the commercial movement of infected, generally asymptomatic, planting material represents the most significant route by which the pathogen has spread on a global scale. Eradication becomes difficult or impossible once the bacterium is established in local soil and irrigation systems, and therefore, strict quarantine regulations are applied in many countries. For instance, *R. solanacearum* R3bv2 is considered to be a quarantine pathogen in Europe and Canada and is listed as a select agent in the U.S. Agroterrorism Protection Act of 2002.

A genomic basis was sought to develop specific assays for cool temperature-adapted strains of R3bv2 of *R. solanacearum*. Draft genome sequences of *R. solanacearum* R3bv2 NCPPB909 and CFIA906 were decoded using paired-end Illumina HiSeq and MiSeq sequencing technology with TrueSeq V3 chemistry (National Research Council Canada, Saskatchewan, Canada). Totals of 4,998,722,502 bp and 924,954,522 bp were obtained from 300-bp inserts to provide approximately 84× and 105× genome coverage for NCPPB909 and CFIA906, respectively. After quality checking and initial *de novo* assembly using Velvet assembler (6) and SPAdes assembly (7), the draft genome size for NCPPB909 is 5,252,833 bp consisting of 220 contigs with 66.8% G+C content, and the draft genome for CFIA906 is 5,037,039 bp consisting of 165 contigs with 66.7% G+C content. Annotations conducted on the RAST server using the Glimmer 3 option (8, 9) predicted 4,937 and 5,025 protein-coding genes, including 53 and 63 noncoding RNA genes for NCPPB909 and CFIA906, respectively. A number of predicted virulence related factors, phage-related loci, motility, and chemotactic genes were identified in the genome, which may enhance or trigger pathogenicity in specific environments.

Further analysis of these two strains will specifically focus on low-temperature adaptations and its correlation with pathogenicity characteristics of R3bv2 strains. The analysis will provide further insight into the virulence, functionality, and plant/pest interactions of this widely distributed and regulated plant pathogen.

### Nucleotide sequence accession numbers.

The draft genome sequences of *R. solanacearum* strains NCPPB909 and CFIA906 have been deposited in GenBank under the accession numbers JNGD00000000 and JNVP00000000. The versions described in this paper are JNGD01000000 and JNVP03000000.
